# Future perspective of planning child guidance services in India

**DOI:** 10.4103/0019-5545.44744

**Published:** 2008

**Authors:** P. C. Shastri

**Affiliations:** President, Indian Psychiatric Society and SAARC Psychiatric Federation, Hon Psychiatrist - B.Y.L.Nair Hospital (Rtd.), Department of Psychiatry, T. N. Medical College (Rtd.), Mumbai

Human behavior has bio-psycho-socio-cultural base.[1] Behavior development is primarily based on genetic maturation, learning, and socialization pattern. Child is active and interactive with his environment during various developmental phases, which makes dynamic understanding of large number of issues centered around mental health of child a very difficult task. It is not surprising that very little work has been done in this area of mental health of children in the world, more so in India. Varied expressions of the same may be “Child is the discovery of the century,” “Biggest discovery of the century is our knowledge of extent of our ignorance,” and “I knew various theories of child mental development, now I know many children with the distinct problem of their own.[2]”

India presents a unique case in terms of its large population and 50% of them are children and adolescents; characterized by heterogeneity in respect to physical, economical, social, and cultural conditions. India's population of 1050 millions makes 16% of the world population, 68% of which is living in the villages.[3]

India is secular with various languages, cultures, and religions. It has 179 languages, 544 dialects, and 1942 mother tongues; with 148 mediums of instruction at school level. This kind of complex and multifaceted country makes formulation of national policies, programing, and planning very difficult.

The Nation's children are our supremely important assets. Their nature and solitude are our responsibility. Children's programs should find a prominent part in our national plans for development of human resources, so that our children grow up to become competitive citizens. Equal opportunities of development to all children during the period of growth should be our aim, for this would serve our larger purpose of reducing inequality and ensuring social justice.

It shall be the policy of the state to provide adequate services to children, both before and after birth, and through the period of growth to ensure their full physical, social, and mental development. States shall progressively increase the scope of such services, so that, within a reasonable time, all children in the country enjoy optimum condition for their balanced growth.

India is a country of children, adolescents, and young adults. It is not only the mental health needs of this 60% of the young population that we are addressing to, but also future generation's mental health. Prevention is better than cure. It is well known that adult psychopathology and mental health problems are only an extension of child mental health problems and continuum in psychopathology. It is not surprising that mentally disturbed parents produce mentally disturbed children, who in turn, will again grow into mentally disturbed adult.[4]

According to the World Mental Health Rights of Mentally ill (1998) - depression, suicide, alcoholism, and psychosis comprise of 75% of mental illnesses and hence they need special attention. Adult influence on child mental health is considerable. For the child this adult can be the protector, provider, legal guardian, custodian, or caretaker.[5]

A good quality-of-life for every child includes good housing, health services, financial stability, family environment, social network, practical coping skills, etc. Ninety percent of the children in India have a very poor quality-of-life.

Child and adolescent mental health, which is future of our country, is given inadequate attention. As overall development of any country is dependent on positive mental development of its children, it is definitely the challenge of the day to cope up with poverty, malnutrition, illiteracy, poor health, and hygiene that is crippling millions of children in India.

Changing structure of the family, modernization, westernization, industrialization, globalization, and urbanization have negatively influenced child mental health. Incidences of mental health problems are on increase (278% increase in pedophilia). Depression and suicide have increased three to four folds in large number of states in India. Post-traumatic mental disorders have shown phenomenal rise. During this decade far more children have been killed and disabled than soldiers. Mental health problems in children affected with riots, bomb blasts, and natural catastrophes are perpetually ever increasing in number. Alcohol and other drug abuse in children have increased ten fold. The recent data indicate the overall prevalence of mental and behavioral disorders among children was 12.8%. This amounts to 66 million children needing special care, attention, and guidance.[5]

In India, child mental health services have been neglected for the last 57 years. National Mental Health policy makers (2003) have practically nothing on their agenda as far as child mental health policy and planning are concerned. It is a sorry state of affair. In last 67 years from 1937 when first child guidance clinic (CGC) was introduced till 2003, NIPCCD study located only 164 CGCs - roughly only two CGCs a year and that too only in metro and mega cities. All these child guidance movements and mental health activities and services have been initiated and sustained by efforts from NGOs.

If we target one CGC per one lakh population we will need 10050 CGCs in the entire country. We need to be innovative so that mental health services for children are possible both in rural as well as urban sectors. For this, question of child mental health should be treated as entirely a special subject and there should be separate units and personnel working with and for the children. An autonomous body with an interdisciplinary perspective has to be created, which should be responsible for the development of child mental health services in an organized manner. Decentralization of child mental health services is a must. Health of people should be in the hands of people. An innovative program and plan should consider important sociocultural dimension and dynamics during implementation.

Following basics in child mental health should not be compromised to make it effective and successful:

1. Affordability
2. Availability
3. Accessibility
4. Acceptability
5. Appropriate technology

The multidisciplinary and multiple services should be at one center. It is a well-known fact that India does not have enough specialists to manage mental and behavioral disorders. Nevertheless, efforts should be made to have one such team in each district. Centrally placed team should be able to manage all the peripheral CGCs in the district with the help of one community based team that finally reaches the people at their doorstep. With roughly 500 districts in India we will need 1000 multidisciplinary teams. Initially, all those who have been in the child guidance movement and child mental health services should be invited to participate in training program for the trainers at district level and at periphery.

Experts in the field of child mental health services should be invited to innovate services and training program for this neglected area. Child mental health issues of our country need to be addressed with intensity and urgency. This should incorporate cross-cultural, multilingual, and multiregional requirements. Child mental health program should cover prevention, early identification, intervention, rehabilitation, and integration. It is vital that child mental health program should have local contexts with due regards for local culture and religion.

The amount spent on mental health in India remains only 0.83% of the total budget. Within this limited budget the share of our children is further marginalized. There is no specific allocation for child mental health services in tenth five-year plan. Government contribution to improve the state of affairs is negligible. Grants are reducing every year in spite of increase in cost and population. NGO's work is hindered by need to get licenses for voluntary work.[3]

India has been signatory to all the resolutions including the latest passed on 1^st^ January 1996, which states that every child will have equal opportunities, protection of right, and full participation (The Persons with Disabilities act, 1995). After five decades of independence we have resolved to help the Indian child but only “if within the economic capacity” of the state and central government. Child has never been given the minimum essential in last five decades. It is not surprising that under the “minimum need program” during last ten years, outlays and expenditure under the ‘Mental Health Sector’ has been very insignificant and underutilized. This also portrays a negative picture of the needs in this sector and hinders expansion of financial needs of the same by the future planning bodies.

The schism of “NO FUND” or unutilized or underutilization of fund is typical of India in area of child mental health. It is not surprising to know that none of the states have achieved the national norms of population to be served by community health center (CHC) in last 50 years.

In spite of the dream to achieve “Health for All” by 2000, present emphasis is not on ‘REACHING THE UNREACHED’. Government has not sanctioned a single subcenter in last 14 years (since 1990). Present emphasis is on consolidation of existing services and no expansion. The policy makers have resolved to have qualitative and not quantitative improvement in mental health services and/or general health services in India. It is a stark contrast to have a target and to not do anything to achieve the target.

Facilities should be provided for special treatment, education, and care of children who are physically handicapped, emotionally disturbed, or mentally challenged.

Ten percent of the child population is in need of special care and treatment. Only one in 100 get some. It is high time we reach out to the rest 99% of the child population that is being unattended by any agency. Children with borderline intellectual functioning and various learning, speech, visual, and hearing difficulties are conservatively estimated to be 20% of the total child population. These 114 million children have no facilities even in the urban areas.

In spite of three-fold increase in grants, release for the children in need of special care, who are the beneficiaries, has come down to half. It means more funds for more staff members and service providers and less for the target group that needs these services. To be precise, 90% of expenses go to the staff and only 10% expenditure is on the child. Ideal planning should consider 70% of the expenditure on the child.

Special groups like blind, deaf, mentally challenged, cerebral palsy and multiple handicaps have very specific requirements. It is also important to know that large number of NGOs run various developmental clinics, CGCs, and school mental health clinics to cater to the specific needs of each disability sector. They should be utilized as a central nodal point for further expansion of considerable child mental health services.

Development clinics not only will fulfill requirements of annual examination and evaluation of each and every child in India but also help in early detection and prevention of a large number of disabilities. ‘Catch them young, as young as you can’, helps in early intervention strategies.

The existing child guidance movement should be a nodal center for further expansion of child mental health services. Following the example of the present team that is working in the CC, a similar multidisciplinary team should also be setup at community level to reach out the unreached in the community.

School mental health program should get maximum attention and help, as large majority of the children can be reached this way. Thirty percent of the school-going population is in need of mental health care. It is vital that the service model and mental health service delivery system should get top priority to school mental health.

Child population is not homogeneous. Large number of children have no home, no school, and no family. They can be in orphanages, destitute homes, beggars' homes, juvenile homes, rescue homes, and remand homes. They can even be street children. All these groups some how have their own self-help group, one of the motto of such group is “each one teach one” to become self-sufficient. Some of them run their own CGC.

Prostitutes, victims of abuse, traffic, and violence form a special group of children who need entirely different kind of expertise, service, and care.

India is unique. India has largest population of married children below the age of 14 years, which is 14.28% of the total population. This accounts for 43.7 million children in India. These children are unique in their problems as they are minors who are supposed to look after new born and young children. This is a large group that needs to be addressed not only in child guidance but also in group therapy sessions in the community. Family therapy and psychoeducational therapy may be effectively used to help these groups of child population.

It is essential to plan the strategies for such wide, diverse population and create linkages and integrate services in settings where children are already available. Universal education and school for every child would be an excellent ground to integrate mental health services. The schools would then act as safety nets to promote mental health in children and provide timely intervention when required.

Biggest challenge is in developing Human Resources who work in the area of child mental health. From grass-root level, local proximal worker to a large number of paramedical and medical experts need to be committed and devoted. A sustained involvement in this field will need lot of innovation. We will have to give license to hands that care. It is a distant dream in India to have a child development program, which makes the child a healthy person, partner, and parent. Mental health professionals in child development have lot more to do before one can think of ideal care for children by 21^st^ century clinician.

## References

[1] Backman L, Hofsten C (2002). Psychology at the Turn of the Millennium.

[2] Bloom MV, Cutter MN, Davidson R (2000). Genes, environment,and Human Behavior, Biological Sciences Curriculum Study (BSCS) 5415 Mark Dabling Blvd.

[4] Sharma I (2005). Editorial -Mental Health Care - Sensitization to Children's Needs, J Indian Assoc Child Adolesc Ment Health.

[5] World Health Organization, The World health report (2001). Mental health: new understanding, new hope. Chapter 2: Burden of Mental and Behavioural Disorders.

